# Estimating Three‐Dimensional Posture to Correct Drone‐Based Measurements of Free‐Swimming Marine Mammals: An Application to Dugongs in Shallow Waters

**DOI:** 10.1002/ece3.74446

**Published:** 2026-10-20

**Authors:** Chiaki Yamato, Helen L. Sneath, Janet M. Lanyon

**Affiliations:** ^1^ School of the Environment The University of Queensland St Lucia Queensland Australia; ^2^ Akkeshi Marine Station, Field Science Center for Northern Biosphere Hokkaido University Akkeshi Hokkaido Japan

**Keywords:** 3D model, body size, drone photogrammetry, marine mammals, oblique drone imagery, pose estimation

## Abstract

Drone‐based measurements of free‐ranging aquatic wildlife are inevitably affected by posture variations, yet this issue has remained largely unaddressed. A widely used approach is to visually inspect body curvature based on overhead shots and exclude animals exhibiting strong body curvature, which may result in a substantially reduced dataset and inconsistent quality control. We developed a drone‐based method of estimating body measurements of dugongs using dual drones (positioned vertically and obliquely relative to the animal) whilst considering body posture (Method_dual_). We reconstructed 3D coordinates of the body's dorsal line and scaled these based on altitude obtained with Real‐Time Kinematic measurements. Estimation error was evaluated using 25 free‐swimming dugongs for which direct body measures were obtained out‐of‐water and compared with a conventional method based only on overhead shots (Method_single_). General surfacing posture of animals was further examined using 188 wild dugongs. Method_dual_ achieved significantly smaller absolute errors in body length (BL) than Method_single_ both when the body was relatively curved (0.039 ± 0.033 m or 2.0% ± 1.6% of BL versus 0.114 ± 0.084 m or 4.8% ± 3.8%) and straight (0.033 ± 0.028 m or 1.3% ± 1.1% versus 0.05 ± 0.042 m or 1.5% ± 1.2%). Method_dual_ provided reliable estimation even when the body curve ratio (ratio of apparent BL from the overhead shot to the posture‐corrected 3D BL) was lower than 0.919 (i.e., body curve is stronger), whereas Method_single_ did not. The mean body curve ratio of free‐swimming animals was 0.926 ± 0.049 (0.751–0.992; *n* = 188) and 31.9% had a body curve ratio below 0.919. The proposed method enables data collection from surfacing events that would otherwise have been discarded as poor quality, which may be particularly useful when encounter rate is low. This method also helps establish quantitative thresholds for data inclusion.

## Graphical abstract

Drone‐based measurements of free‐ranging aquatic wildlife are inevitably affected by posture variations, yet this issue has remained largely unaddressed. We developed a drone‐based method of estimating body measurements of dugongs using dual drones (positioned vertically and obliquely relative to the animal) whilst considering body posture. The proposed method is applicable to a wide range of free‐swimming mammals and enables data collection from surfacing events that would otherwise have been discarded as poor quality and also helps establish quantitative thresholds for data inclusion.

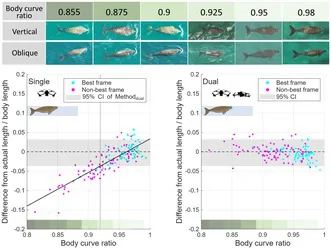

## 1 Introduction

Morphometric traits of wildlife are fundamental parameters for assessing body condition, population structure, and population dynamics, serving as key tools for ecological and conservation studies (Green 2001; Schulte‐Hostedde et al. 2005). In recent decades, drone photogrammetry has gained popularity for measuring aquatic animals that are challenging to find, approach, or capture (Johnston 2019).

The desirable accuracy for morphometric measurements of aquatic animals depends on the research question and size of the target species. For example, assigning three age categories in bottlenose dolphins ( *Tursiops truncatus* , 2–3 m total length) requires reliably resolving ~40 cm size bins; finer categorization demands greater accuracy (Vivier et al. 2023). Assessing relative body condition across or within populations can be demanding. Body condition index as an indicator of energy stores is often derived from body width profiles standardized by total length (Bierlich, Hewitt, et al. 2021). In the case of gray whales ( *Eschrichtius robustus* , 7–14 m total length), the standard deviations of maximum widths within size/maturity categories are 10–20 cm (Lemos et al. 2020), indicating that biologically meaningful differences of morphometric traits can be subtle even in large‐bodied species. Seasonal differences in mean maximum width (i.e., axillary width) estimated using drone photogrammetry were only 2.5 cm for franciscanas ( *Pontoporia blainvillei* ; 0.9–1.6 m total length; axillary width: 18 ± 2 cm) and 4.0 cm for Guiana dolphins ( *Sotalia guianensis* ; 1.0–2.0 m total length; axillary width: 29 ± 4 cm) (de Oliveira et al. 2023).

One of the central challenges for drone photogrammetry of aquatic animals has been the establishment of scale (i.e., ratio between pixel and real‐world lengths) when measuring body size of target animals. One solution is to film an object of known length at multiple heights during each flight and regress the scale against sampling altitude on a per‐flight basis. This approach has reduced the measurement error for a 1‐m floating object from 0.34 to 0.17 m (Burnett et al. 2019). Another solution is to fit a drone with a Light Detection and Ranging (LiDAR) unit, which measures pulsed laser light reflected from the target to determine its distance (Dawson et al. 2017; Bierlich et al. 2024). This unit has measured a 2.7 m floating object with an error of 2.7 ± 4.9 cm (Dawson et al. 2017) and a 1.5 m on‐land object with an error of 0.7 ± 0.5 cm (Christiansen et al. 2018). Another solution is to use drones that employ carrier‐phase measurements (e.g., Real‐Time Kinematic (RTK) or Post Processing Kinematic (PPK) drones), where the positioning accuracy is improved to centimeter‐level by using correction data from a fixed base station with a precisely known location (Martínez‐Carricondo et al. 2023). The recent non‐expensive models offer both nominal centimeter‐level positional accuracy (DJI 2025) and validated centimeter‐level accuracy of 3‐dimensional (3D) maps they produce (Fraser et al. 2022).

Measurements of free‐ranging wildlife are inevitably affected by posture variations, yet their effects on accuracy have remained largely unevaluated, primarily due to the challenges in obtaining ground‐truthed measures from living wild aquatic megafauna. Ramos et al. (2022) estimated the total body length of a captive 3.14 m Antillean manatee ( *Trichechus manatus manatus* ) with estimation errors of −0.19 m (6.1%), −0.16 m (5.1%), and −0.03 m (1.0%) with filtered images (i.e., relatively straight posture) using a LiDAR drone. They also reported an error of 21.3 ± 11.6 cm or 8.2% ± 3.8% using a non‐LiDAR drone. A study of bottlenose dolphin achieved centimeter‐level accuracy (error of 3.3% ± 3.1% or 8 ± 8 cm) by estimating total body length from the blowhole‐to‐dorsal‐fin distance (BHDF) (Vivier et al. 2023). This anterior region represents approximately one‐third of the body length (Vivier et al. 2023) and typically comes above the water surface upon breathing, making it less affected by body curvature during surfacing or extremities (e.g., tail fluke) obscured by water. Such an approach is unfeasible for species that lack distinct features along the body centerline (e.g., dorsal fin) including sea cows (dugongs and manatees) and finless porpoises. Most drone photogrammetry studies of aquatic mammals include animals showing only minimal body curvature. A widely adopted approach scores each image for camera focus, body posture, and body measurability, and excludes images of insufficient quality (Christiansen et al. 2018). After this screening, for example, Christiansen et al. (2018) retained 1118 of 2890 photographs. When different photographs from the same individual have been obtained, the precision (repeatability) of body measurements has been confirmed to not affect the final model parameters that address their ecological questions (Christiansen et al. 2016, 2018). Previous studies retained 38%–94% of data after visual screening of image quality and posture (Christiansen et al. 2016, 2018; Lemos et al. 2020; Yamato et al. 2024). For populations with low encounter rates, a posture issue may result in the absence of usable data. Correcting measurements via estimated posture may help improve success rates of sampling, expand datasets, mitigate sampling bias, and establish quantitative thresholds for data inclusion.

Pose estimation studies are most advanced in humans. Successful approaches include training a generative model with thousands of 3D scans of individuals in various poses (joint locations) and subsequently fitting this model to 2D image data (Loper et al. 2015). One study applied this model to estimate the posture of terrestrial quadrupeds using oblique drone footage, although ground‐truthing was suggested as a future step (Shukla et al. 2024). Applying such a model to marine mammals would be even more challenging due to the lack of distinctive body landmarks (such as limb joints), and the invisibility, ambiguity, and distortions of these landmarks caused by water. A simpler approach estimates the 3D coordinates of arbitrary points using multiple cameras whose precise relative positions and orientations are calculated at the beginning and/or end of filming (Karashchuk et al. 2021). Pose estimation of free‐ranging wildlife that cannot be recorded from a fixed location is more challenging. Where it is possible to approach the animal, as in the case of fish, 3D locomotion has been estimated using two cameras mounted on an arm whose relative positions and orientations were precisely calibrated before and/or after sampling (Décamps et al. 2017). To obtain multi‐angle footage of free‐swimming aquatic megafauna in natural environments, it is more realistic to use drones. To our knowledge, no studies have attempted pose estimation of free‐moving aquatic wildlife using multiple drones. The system we describe here estimates 3D coordinates of body dorsal lines using two drones, and scales them based on altitude obtained with RTK drones with corrections using a reference object.

The dugong ( *Dugong dugon* ) was selected as the target species (Figure 1). As the only fully marine herbivorous mammal, the dugong plays a unique and vital role in coastal ecosystems, yet it is now threatened throughout its range (Marsh et al. 2011, 2025). Because many dugong populations occur in remote areas and/or in turbid waters, and are small, low density or unpredictable (Marsh et al. 2025), drone‐based photogrammetry represents one of the few feasible methods for obtaining morphometric data to assess growth stage, population structure, and body condition (e.g., Goudalier et al. 2025; Patel et al. 2025; Yamato et al. 2024). Due to the lack of distinct features along body centerline, drone‐based measurement of dugongs is particularly affected by body curvature.

**FIGURE 1:**
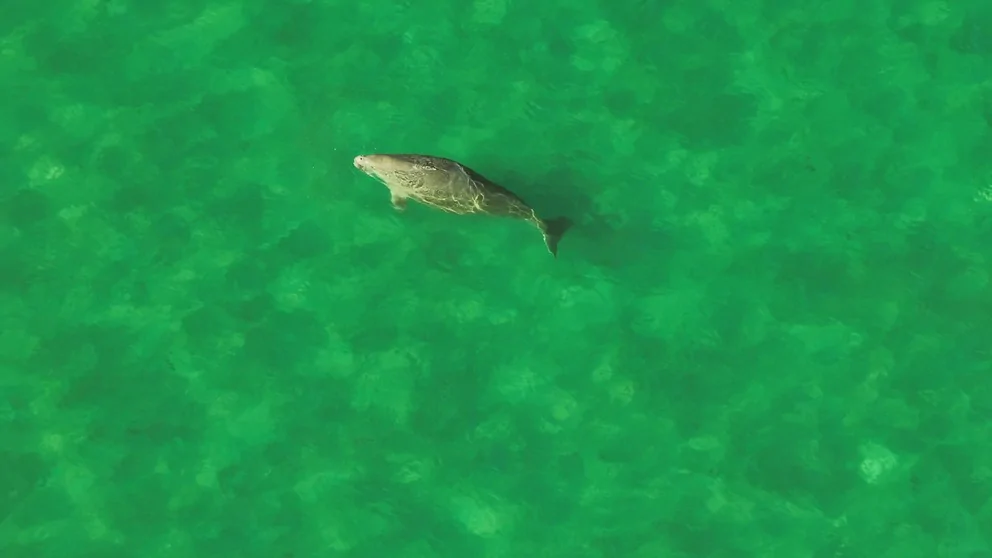
Dugong ( *dugong dugon* ).

Our aim was to develop a drone‐based method of estimating body measurements (including body length) of dugongs, accounting for their 3D posture. Estimation error was evaluated using free‐swimming dugongs for which direct body measures were obtained. It was anticipated that both conventional and proposed methods would perform well when the animal's body was straight, and only the proposed method would maintain good performance when the body was curved. We determined this threshold for the degree of body curvature and reported the proportion of free‐swimming dugongs exceeding this threshold to evaluate how the proposed method could contribute to expanding and improving the reliability of datasets.

## 2 Materials and Methods

### 2.1 Data Collection

Drone footage was successfully obtained from (1) free‐swimming dugongs for which direct body measures were obtained as part of a health assessment program, in which wild dugongs were temporarily captured and removed from the water for comprehensive medical examinations (*n* = 25) (Lanyon et al. 2010) and (2) a random sample of free‐swimming dugongs to examine general variation in posture (*n* = 188). Health assessment sampling occurred in November 2024, March 2025, and August 2025, whilst random sampling occurred in November 2024 and May 2025, all in Moreton Bay, Queensland Australia. Footage was collected in relatively calm conditions (Beaufort sea state 0–3, < 10 knots). Filming always commenced when dugongs were in shallow water (1–4 m), although footage was occasionally recorded over deeper water if the dugongs moved.

Direct body measurements were taken with a tape measure from stationary, recumbent, prone dugongs on the deck of a research vessel. Measurements included straight body length (measured in a straight line from snout to fluke notch), curved body length (along the body contour from snout to fluke notch), fluke width, and straight length from snout to nostril. While snout–fluke notch length has served as the standard body size measure for dugongs (Lanyon et al. 2002), this study also tested whether the use of all possible markers along the body centerline (i.e., using the central point of nostrils (nares) as the start point of body length and/or using the narrowest and closest point of the peduncle to the fluke as the endpoint) mitigated the effect of body curvature along the body axis. Because straight snout–nostril lengths were not obtained for five animals, these animals were excluded from the analysis evaluating of body length measurements using alternative extremities. Each animal was marked with a waterproof Markal Paintstick crayon (Dy‐Mark, Qld, Australia) at 10 evenly‐spaced points along the central dorsal line from snout to fluke notch so as to identify the body dorsal line in drone footage. All animals were apparently healthy with no clearly visible spinal ridge.

Because there was likely uncertainty in identifying the peduncle (i.e., the narrowest and closest point to the fluke) during direct measurements, some measurements were also taken post‐field. Multi‐angle videos were taken of the dugong lying prone on the boat deck from the greatest practicable distance (approximately 1–1.5 m) using a handheld camera (GoPRO HERO12), to generate a three‐dimensional model of each dugong with a photogrammetric software (Metashape Professional Edition v1.5.4, Agisoft LLC, Saint Petersburg, Russia) following Setiyadi et al. (2021). Curved length from snout to nostril and from peduncle to fluke notch were obtained from the 3D model, scaled using the known accurate fluke width measured on deck. The peduncle–fluke notch length measured on deck often differed by several centimeters from that obtained from the 3D model scaled with fluke width. The absolute estimation error of 3D models of a stationary object (a crate measuring 30 × 30 × 110 cm) generated using the same method was 0.59 ± 0.43 cm (*n* = 6; range: 0.1–1.1 cm). Two models of the object placed at two different field locations were generated, scaled using a reference length of 29.8 cm, and validated against known lengths of 10.7, 30.2, and 30.2 cm. Consequently, the length from the 3D model was used because (1) the 3D model error was considered acceptable and (2) the models allowed careful inspection and alignment with drone‐based measurements. Because 3D models were not obtained for five animals due to either of data loss or insufficient footage, measurements for these animals were obtained from drone footage taken of the animal on deck or upon release (when the animal rolled at water surface and the snout and nostrils were visible). Drone footage of each health assessment animal was taken upon their release.

Two small drones (Mavic 3E, DJI, Shenzhen, China) monitored each dugong for approximately 10 min from 40 m altitude at 7× optical zoom, using a 35‐mm‐equivalent focal length of 162 mm and yielding an empirical ground sampling distance (GSD) of 2.4 mm/pixel (see below and Section A1 in Appendix for details). The video was recorded at 30 frames per second at 3840 × 2140 pixels throughout the entire flight. Before takeoff and after landing, chopsticks were tapped in front of the facing cameras for synchronization. Before and after observation of the focal dugong, each drone filmed a reference object of known length at the water surface (a vessel duckboard or a buoy) from an altitude of 40 m for calibration. The first camera (Camera V) captured the animal vertically from directly above, and the second camera (Camera O) from an oblique angle of approximately 45°. A pilot study was conducted to compare footage obtained from oblique angles of 30°, 45°, 60°. The 45° angle was selected to balance adequate horizontal spacing between drones of 40 m with sufficient visibility of the animal's body extremities (snout and fluke). Both drones collected footage of the reference object and dugong at 7× optical zoom. The drones corrected their positions in real time using reference points with known coordinates measured using a Global Navigation Satellite System (GNSS) receiver (DG‐PRO1 RWS, Bizstation Corp., Nagano, Japan) on land. Onboard internet connection for the drones was established with an antenna (Starlink Mini, Space Exploration Technologies Corp., CA, USA) attached on the bimini roof or on the upper‐deck guardrail of the vessels. On‐land internet connection for the GNSS receiver was provided via a mobile Wi‐Fi router.

Following a review of all drone footage using Windows Media Player, the timings of the chopstick strike, filming of the reference object, and each dugong surfacing event (breath) were recorded. All subsequent analyses were performed using custom‐developed code in MATLAB R 2025a (MathWorks Inc., MA, USA). Mavic 3E exported flight logs corresponding with each video file. Altitude was recorded at millimeter‐level resolution and latitude and longitude to six decimal places (i.e., sub‐meter‐level resolution), all at 30 frames per second. Time‐synchronized video clips and flight logs for calibration and all breaths were generated by matching the frames at which the chopsticks were struck.

### 2.2 Estimation Performance of Body Length

The estimation error of body length was examined using ground‐truthed dugongs (*n* = 25). The drone video clips in which the animal was out of frame were excluded. Three surfacing events with breaths were then randomly selected for each animal.

From each breath, the *best frame* (at timing t) was selected. The best frame was defined as the image in which the animal's body was the most parallel to the water surface, and both extremities of body length (snout and fluke notch) were the most visible. The *non‐best frames* (at t±1s and t±2s) were then examined to test the applicability of the proposed method to more curved body postures. The non‐best frame was included if (1) a part of the body was at the water surface (as this condition can always be expected when targeting breathing animals) and (2) extremities were visible (see example in Figure 2). These datasets were used to assess the robustness of the estimates different body postures. In addition, for evaluation of body lengths using alternative extremities (i.e., nostril, peduncle), the best and non‐best frames were selected when (3) nostril and peduncle were visible (Figure 2).

**FIGURE 2:**
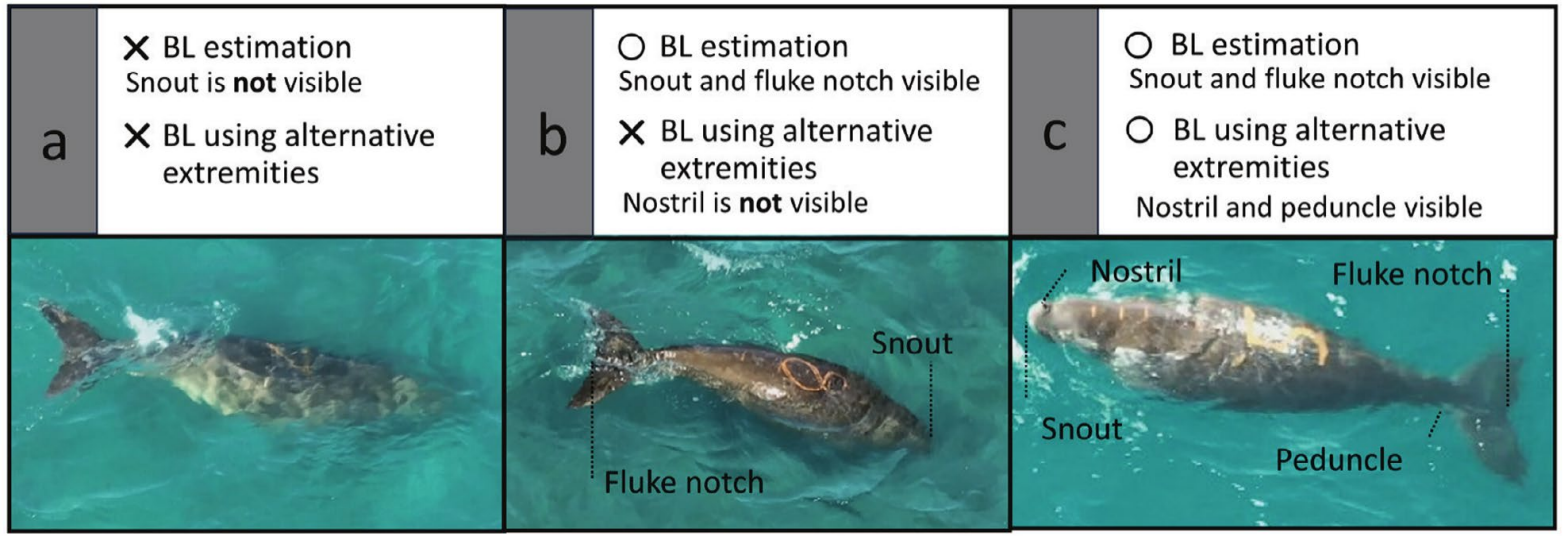
Examples of excluded and included drone images of dugongs based on image quality. (a) Data excluded from both evaluations; (b) Data included in the evaluation of body length (BL) estimation, but excluded from the evaluation of BLs using alternative extremities; (c) Data included in both evaluations.

Lens distortion in each frame was corrected using the Single Camera Calibrator App in MATLAB R 2025a (see A2 in Appendix A for details). In each frame, (1) the central body dorsal line, (2) the boundary between the in‐water and out‐of‐water area along the body dorsal line, and (3) nostril and peduncle were annotated (Figure 3 and Figure A1). The 3D coordinates of the body dorsal line were estimated by tracing rays from the drone camera positions using camera (gimbal) orientations recorded in the drone log (Figure A2). The apparent intersection of the viewing ray of Camera O with the viewing ray of Camera V passing through the animal's dorsal line was first computed under the assumption of straight light paths in air. For underwater portions of the body dorsal line, points were corrected for water‐surface refraction by projecting each apparent point onto the water plane and computing the refracted ray direction using Snell's law (Figure A3). Body roll was not considered, as no apparent roll was observed during surfacing throughout the study.

**FIGURE 3:**
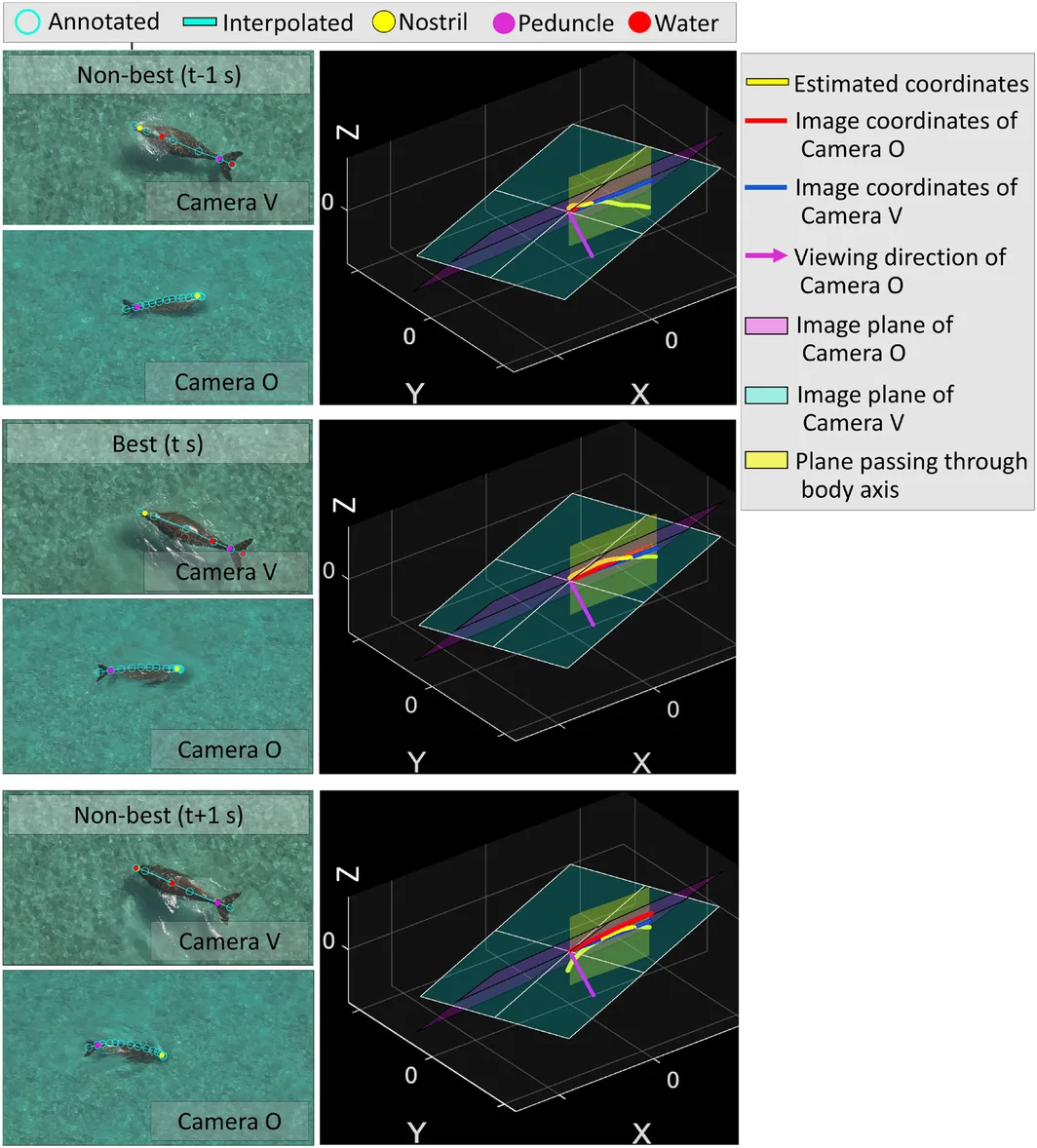
Examples of annotated drone image of dugong and estimated body dorsal line. In the right inset, the image plane and image coordinates are transformed so that starting position of body dorsal line aligns with the origin. The proposed method estimates the total body length (BL_dual_) as the 3D length of the body dorsal line (yellow line), whereas the existing approach estimates the length obtained from overhead shots (blue line, BL_single_).

The lengths of the body dorsal lines were calculated. As an existing method measures body lengths seen from directly above the animal, the length of the dorsal line in Camera V (BL_single_) was compared with the length of the reconstructed 3D dorsal line (BL_dual_). The method used to obtain BL_single_ is hereafter referred to as Method_single_ and the proposed method used to obtain BL_dual_ as Method_dual_ (as in “dual drones”). The pixel length was converted into an actual length using (1) the real‐world and pixel lengths of the reference object filmed before and after each flight (A3 in Appendix A), (2) the real‐world and pixel lengths of a reference object filmed from a directly measured camera‐to‐object distance prior to the field survey (2.4 mm/pixel; A1 in Appendix A), and (3) the drone altitude recorded at the time of reference measurement and body measurement (A6 in Appendix A). The estimation error was then compared between Method_dual_ and Method_single_ using a paired *t*‐test, and among body length measures—(1) snout to fluke notch, (2) nostril to fluke notch, (3) nostril to peduncle, and (4) snout to peduncle—using a one‐way repeated‐measures ANOVA, followed by paired *t*‐tests for complete cases with *p*‐value adjusted for multiple comparisons. The directly measured straight body length was used as a true value for BL_single_, whereas the curved body length was used as that for BL_dual_. The body curve ratio, defined as follows: was calculated for each animal.

$$\text{Body curve ratio}=\frac{{\mathrm{BL}}_{\text{single}}}{{\mathrm{BL}}_{\text{dual}}}$$

A larger estimation error was anticipated when Camera O was not positioned directly beside the dugong (diagonally backwards or forwards) or when body curvature (due to postural changes) increased. This was tested using a linear mixed‐effects model (LMM) with estimation error of Method_dual_ as the response variable. Data from both best and non‐best frames were included. Fixed effects were body curve ratio and camera yaw direction relative to the body axis direction of dugongs, and individual ID was included as a random intercept. The model was fitted using restricted maximum likelihood (REML). Within‐individual variance in estimation error (i.e., precision) across the three breaths and timings (best and non‐best frames) was also reported.

### 2.3 Estimation Performance of Fluke Width

The estimation accuracy of body measurements of underwater animals is inherently limited by water movement, including waves (e.g., ripples and sea waves), water disturbances caused by animal's swimming (eddies), tidal current, by animal breaking the water surface, and by resultant ambiguity of body extremities. Among body parts, fluke width was considered the most suitable for assessing error ranges caused by water movement, as it is free from the errors associated with curvature along the body axis. No apparent body roll was observed in our dataset.

#### 2.3.1 Fluke Width With Clearly Visible Extremities—Effect of Water Movement

Drone footage was obtained for three breaths during surfacing events of two animals filmed under extremely calm water conditions (Beaufort sea state < 1), during which the fluke of known width was almost always clearly visible until the animal surfaced. These data were used to examine temporal variations in the measurement of apparent fluke width that may have been caused by water movement. Fluke width was measured every five frames (frame rate: 30 frames per second) from a 10‐s video clip using Method_single_. Temporal changes in estimation error were then examined.

#### 2.3.2 Fluke Width at Water Surface—Effect of Extremities' Clarity

The effect of clarity of visibility of the body's extremities on apparent body length was examined using the fluke width of all ground‐truthed dugongs. The dataset used in the section “Estimation performance of body length” was used (i.e., three breaths from 25 animals). For each filmed breathing event, the frame in which the fluke was closest to the water surface and fluke tips were most clearly visible was scored for clarity of extremities (fluke tips) using four categories: poor (image out of focus and fluke trailing edge jagged due to water disturbance), fair (fluke trailing edge jagged due to water disturbance), good (fluke trailing edge obscured by water disturbance but not obviously jagged), and excellent (fluke trailing edge clearly visible). The fluke was always positioned slightly below or above the water surface. Differences in absolute error among the four categories were tested using the Kruskal‐Wallis test.

### 2.4 Posture of Free‐Swimming Animals

Body posture of 188 free‐swimming dugongs under natural conditions was quantified to evaluate how the proposed Method_dual_ could contribute to expanding and improving the quality of datasets. For each animal, the best frame of the best breath was selected. The best frame was defined as the image in which the animal's body was the most parallel to the water surface, and both extremities were the most visible. The best breath was defined as the breathing event during which the animal's body appeared most parallel to the water surface.

It was anticipated that both Method_single_ and Method_dual_ would perform well when the animal's body was straight, and only Method_dual_ would maintain good performance when the body was curved. This threshold of body curve ratio was determined to assess how often cases occur where reliable estimation is possible only with Method_dual_. The estimation error of BL_single_ (calculated using ground‐truthed animals) was predicted using a linear regression model with body curve ratio as the explanatory variable, as a linear relationship was observed between the two. The threshold of body curve ratio was determined as the value at which the predicted error of BL_single_ exceeded the 95% confidence interval (CI) of the error of BL_dual_. The 95% CI was calculated as:

$$m\pm t_{0.975,n-1}\times \frac{s}{\sqrt{n}},$$

where *m* is the mean, *s* is standard deviation, and *n* is sample size, and $t_{0.975,n-1}$ is the 97.5th percentile of the Student's *t*‐distribution with *n* − 1 degrees of freedom. Finally, the proportion of animals with a body curve ratio below the threshold was calculated. All means are presented as mean ± SD.

### 2.5 Ethical Considerations

Live wild dugongs were sampled under The University of Queensland Animal Ethics #2023/AE000675, Scientific Permits WA0019236 and P‐SPP‐100788137 and Moreton Bay Marine Park Permits MPP18‐001119‐2 and MPP18‐001119‐3. Drones were operated under Standard Operating Procedure by Civil Aviation Safety Authority. During the random sampling of dugongs, the drones searched for dugongs at a flight altitude of 100–120 m. When a dugong was found, the drones slowly descended and followed it at 40 m, a flight height at which no adverse effects on the dugongs had previously been reported (Yamato et al. 2024).

## 3 Results

### 3.1 Estimation Performance of Body Length

Mean straight and curved body lengths (BL) of dugongs measured on deck were 2.56 ± 0.26 m (range: 1.78–2.96 m) and 2.68 ± 0.27 m (range: 1.95–3.05 m) (*n* = 25). The absolute error (difference between body lengths derived from drone‐photogrammetry and on‐deck measurements) and absolute errors relative to body length of Method_dual_ were significantly smaller than those of Method_single_ both for the best frame (Method_dual_: 0.033 ± 0.028 m, 1.3% ± 1.1%; Method_single_: 0.05 ± 0.042 m, 1.5% ± 1.2%) and the non‐best frame (Method_dual_: 0.039 ± 0.033 m, 2.0% ± 1.6%; Method_single_: 0.114 ± 0.084 m, 4.8% ± 3.8%) (Table A1 and Figure 4).

**FIGURE 4:**
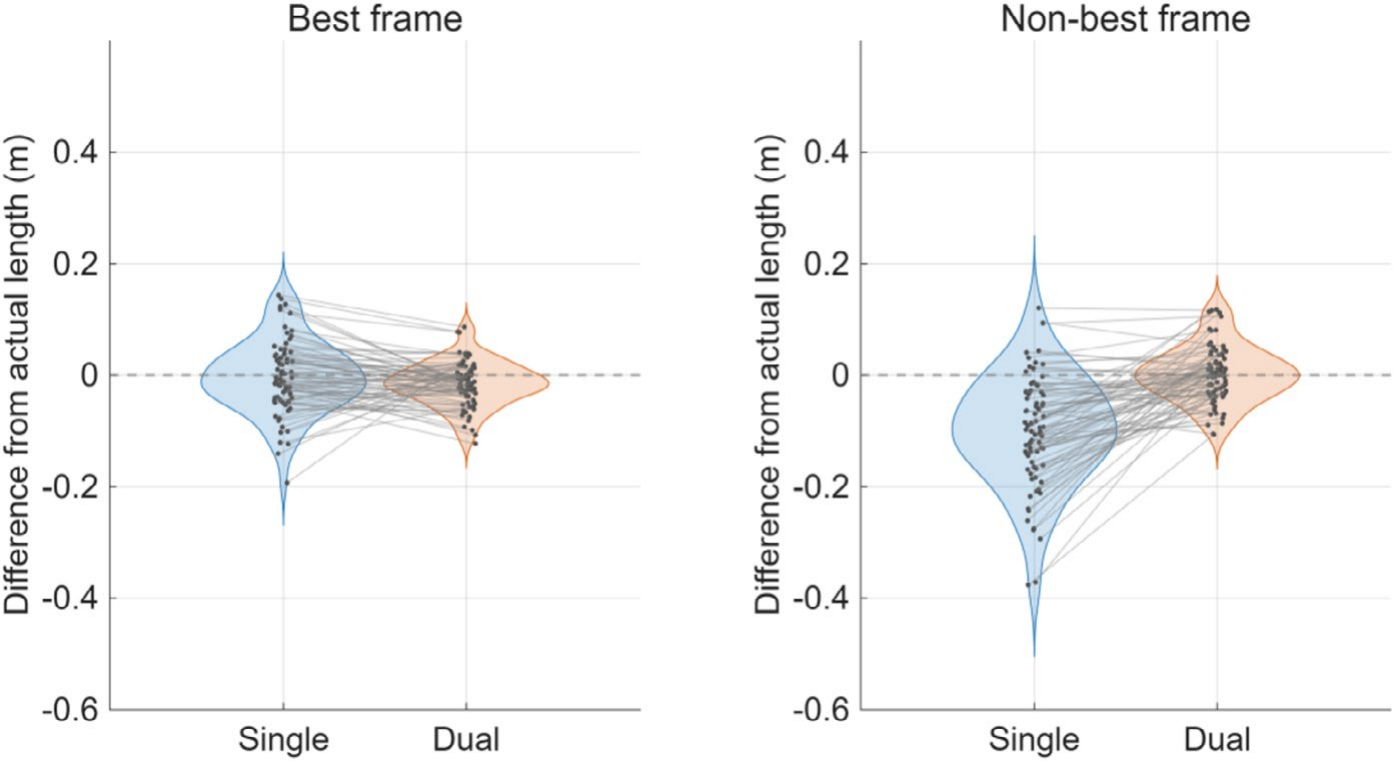
Violin plots of differences between total body lengths (BL) of dugongs derived from drone‐photogrammetry (blue: BL_single_; red: BL_dual_) and on‐deck‐measurements. Data from the same frame are connected with lines.

No evidence was found supporting the effectiveness of using the nostril and/or peduncle as extremities, over the snout and fluke notch (Figure 5 and Table A2). A one‐way repeated‐measures ANOVA revealed a significant effect of body length definitions on estimation error of Method_single_ (*F*_(3,84)_ = 94.6, *p* < 0.001); however, subsequent paired *t*‐tests showed no significant pairwise differences (all *p* > 0.05). For Method_dual_, a one‐way repeated‐measures ANOVA showed no significant effect of body‐length definition on estimation error (*F*_(3,84)_ = 2.08, *p* = 0.11).

**FIGURE 5:**
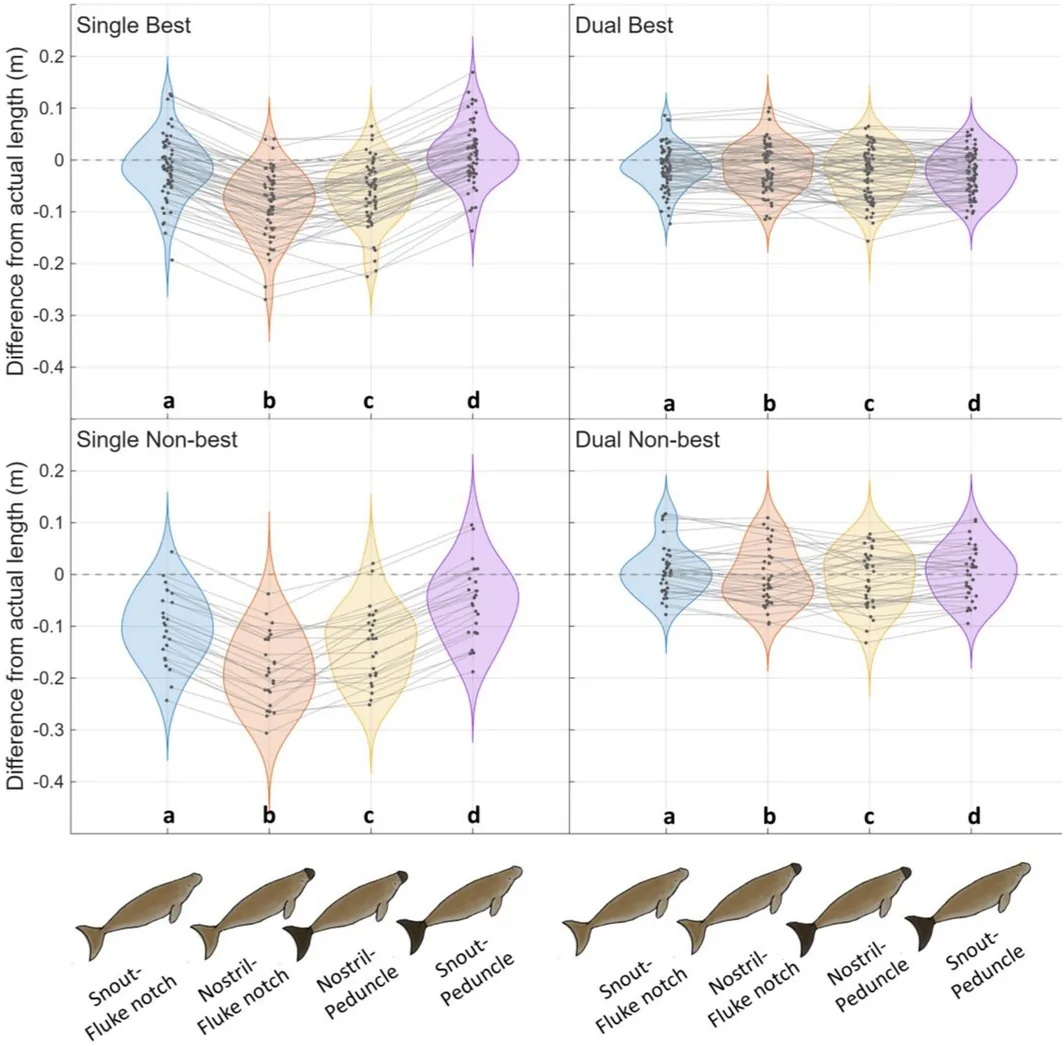
Violin plots of differences between dugong body lengths derived from drone‐photogrammetry and on‐deck measurements. Data from the same frame are connected with a line. Shaded areas of dugongs in the illustrations above each body length definition indicate the body parts that were excluded in the measure of body length.

For both best and non‐best frames, BL_single_ measured from the nostrils had negative estimation errors (−0.085 ± 0.064 m for best‐frame Nostril–Fluke notch length, −0.063 ± 0.061 m for best‐frame Nostril–Peduncle length, −0.179 ± 0.066 m for non‐best‐frame Nostril–Fluke notch length, and −0.132 ± 0.068 m for non‐best‐frame Nostril–Peduncle length; b and c in the left inset in Figure 5). This suggests that the length of the middle body region (distance between nostril and peduncle) was generally underestimated. Because the snout–nostril length was often overestimated as the animals tended to tilt their heads upward while surfacing, the BL_single_ measured from the snout had more positive estimation errors than those measured from nostrils (−0.004 ± 0.066 m for best‐frame Snout–Fluke notch length, 0.016 ± 0.059 m for best‐frame Snout–Peduncle length, −0.081 ± 0.083 m for non‐best‐frame Snout–Fluke notch length, and −0.037 ± 0.075 m for non‐best‐frame Nostril–Peduncle length; a and d in the left inset in Figure 5).

In non‐best frame, BL_single_ measured up to the peduncle had less negative estimation errors than the corresponding lengths to the fluke notch (c versus b and d versus a in the lower‐left inset of Figure 5): −0.133 ± 0.068 m for Nostril–Peduncle length versus −0.179 ± 0.066 m for Nostril–Fluke notch length, and −0.037 ± 0.075 m for Snout–Peduncle length versus −0.081 ± 0.083 m for Snout–Fluke notch length. This is likely because the straight peduncle–fluke notch length tended to be underestimated in the non‐best frame, as the fluke is typically orientated obliquely before a breath.

Unlike Method_single_, Method_dual_ showed no systematic directional change in estimation error when changing the body extremities (the right inset in Figure 4).

The SD of the altitude readings of Camera V was 0.027 ± 0.033 m (*n* = 75, range: 0.004–0.205 m) during t ± 2 s and 0.051 ± 0.080 m (*n* = 75, range: 0.007–0.523 m) over the 10‐s interval including the surfacing event. The differences between the maximum and minimum altitude readings were 0.095 ± 0.118 m (range: 0.010–0.720 m) during t ± 2 s and 0.187 ± 0.275 m (range: 0.020–1.510 m) over the 10‐s interval, respectively. Mean horizontal distance between the two drones was 45.0 ± 3.7 m (*n* = 75; range: 36.3–67.8 m) during t ± 2 s and 45.0 ± 3.8 m over the 10‐s interval (*n* = 75 range: 36–69.2 m).

The mixed‐effects model showed no significant effect of Camera O's yaw direction relative to the dugong's longitudinal body axis on estimation error (*p* = 0.10; Table 1). This suggests that the method is tolerant to observed variation in yaw angle of oblique filming (8.9° ± 6.7°, range: 0.02°–30.4°; a value of zero represents a camera view perpendicular to the body axis, whilst a value of 90° corresponds to a camera parallel to the body axis). A negative effect of body curve ratio on estimation error was statistically significant (*p* < 0.001); however, the predicted errors were centered around zero suggesting the magnitude of the predicted error did not systematically increase across the observed range of curvature ratios (Table 1 and Figure 6). Mean interquartile range of BL estimation errors within individuals across three dives and best and non‐best frames was 0.044 ± 0.018 m (range = 0.014–0.085 m; *n* = 25 animals) (Figure 6).

**TABLE 1:** Fixed effects of the linear mixed‐effects model of estimation error of Method_dual_. The intercept indicates the mean body curve ratio and yaw direction of Camera O relative to dugong's body axis.

Name	Estimate	SE	*t* stat	Df	*p*
(Intercept)	0.4572	0.1173	6.12	150	7.7 × 10^−9^
Body curve ratio	−0.4960	0.1233	−6.23	150	4.6 × 10^−9^
Relative yaw direction of Camera O	1.28 × 10^−4^	5.11 × 10^−4^	0.25	150	0.858

**FIGURE 6:**
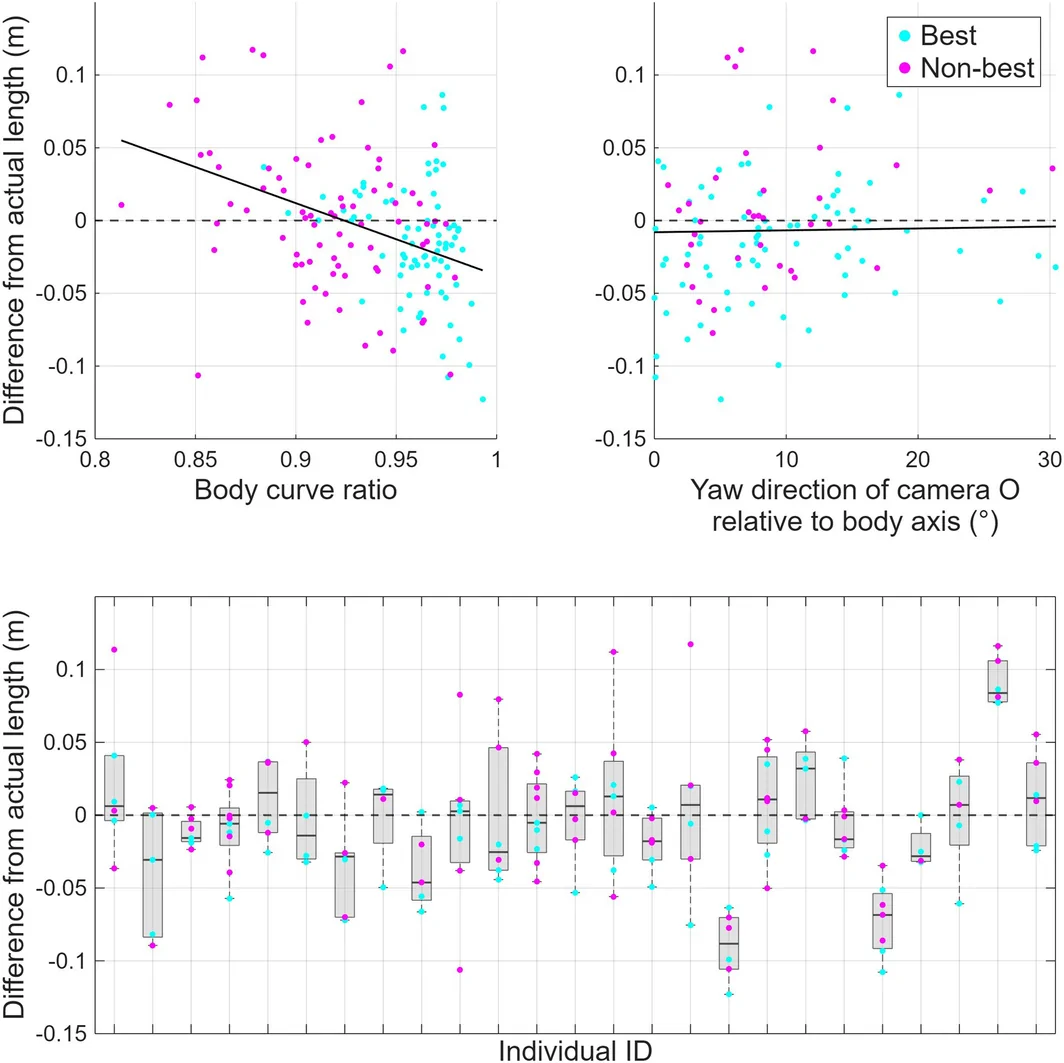
Estimation error of BL_dual_ and explanatory variables of the mixed‐effects model. Top left: Effect of body curve ratio. The black line shows model predictions based on fixed effects. Top right: Effect of Camera O's yaw direction relative to the dugong body axis direction. An *x* value of 0 represents footage taken directly from the side of the dugong. Bottom: Distribution of estimation errors across repeated measurements within individuals.

### 3.2 Estimation Performance of Fluke Width

#### 3.2.1 Fluke Width With Clearly Visible Extremities—Effect of Water Movement

At each surfacing event, before the dugong's nostrils broke the water surface, water disturbances caused by tidal currents and the animal swimming (eddies) were apparent. Even when the fluke tips were almost always clearly visible before breathing, the estimated fluke width varied from its mean by approximately 1 ± 1 cm or 0.4% ± 0.4% of body length (see a in Figure 7). The variation in estimated fluke width mirrored that of pixel length, indicating that the apparent fluke width error was unlikely to originate from instability in drone altitude readings (Figure 7).

**FIGURE 7:**
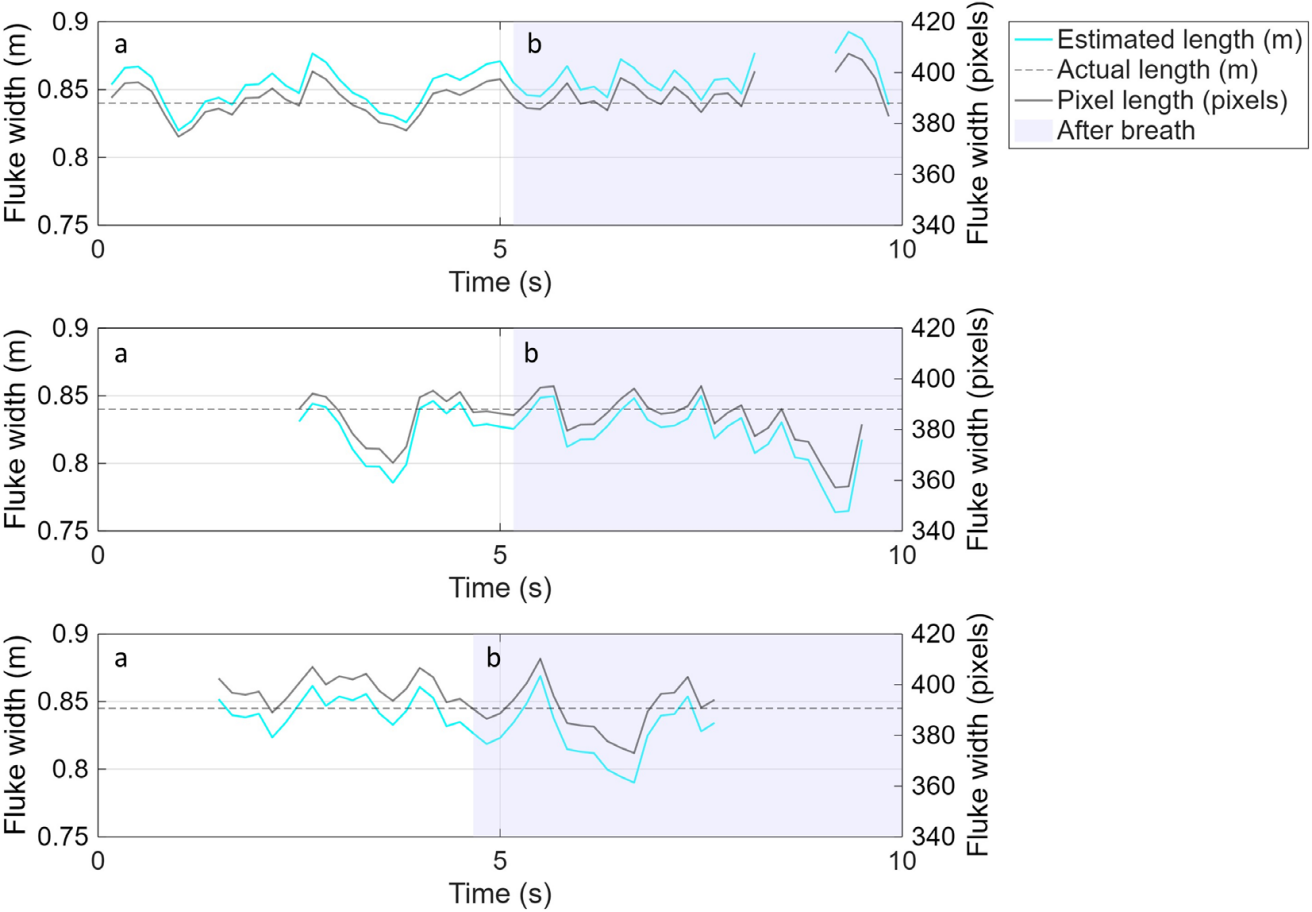
Time‐series changes in estimated fluke width when the fluke tips were almost always clearly visible (a: area with blank background) and when visibility declined as the dugong broke the water surface (b: area with shaded gray background).

#### 3.2.2 Fluke Width Estimation Errors at Water Surface—Effect of Extremities Clarity

The absolute estimation error of 0.023 ± 0.015 m (range: 0.001–0.061 m) or 0.86% ± 0.60% of body length (range: 0.03%–2.3%) significantly decreased with increasing clarity of body extremities (Figure 8, Kruskal‐Wallis test, χ^2^(3) = 9.50, *p* = 0.023); RMSEs (m) were 0.033 (*n* = 15), 0.030 (*n* = 25), 0.0256 (*n* = 15), and 0.019 m (*n* = 20) for “Poor”, “Fair”, “Good”, and “Excellent” frames, respectively. The corresponding median absolute errors were 0.031, 0.025, 0.016, and 0.014 m, respectively.

**FIGURE 8:**
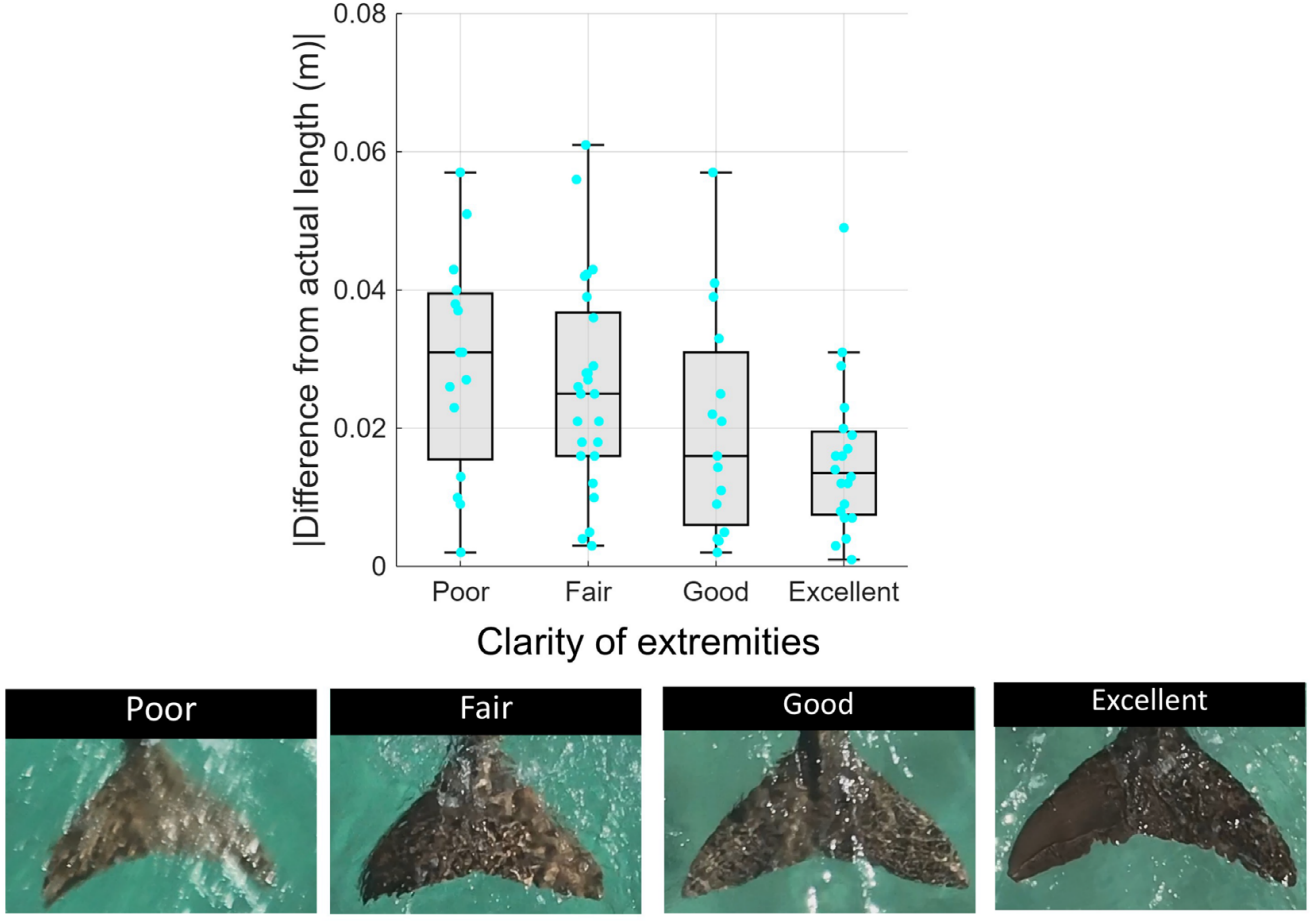
Absolute estimation error of fluke width and clarity of the body extremities. Example image from each quality category is shown below.

### 3.3 Posture of Free‐Swimming Animals

95% CIs of estimation error for Method_dual_ were from −3.7% to 3.2% of body length (Figure 9, right inset). A linear relationship was observed between the body curve ratio and the estimation error of BL_single_ (Figure 9, left inset. *R* ^2^ = 0.705, *p* = 0.868*x* − 0.834), which supports that estimation performance of BL_single_ decreases as the body becomes more curved. The body curve ratio threshold at which the magnitude of estimation error of BL_single_ exceeded the lower limit of 95% CIs of BL_dual_ (−3.7%) was determined as 0.919.

**FIGURE 9:**
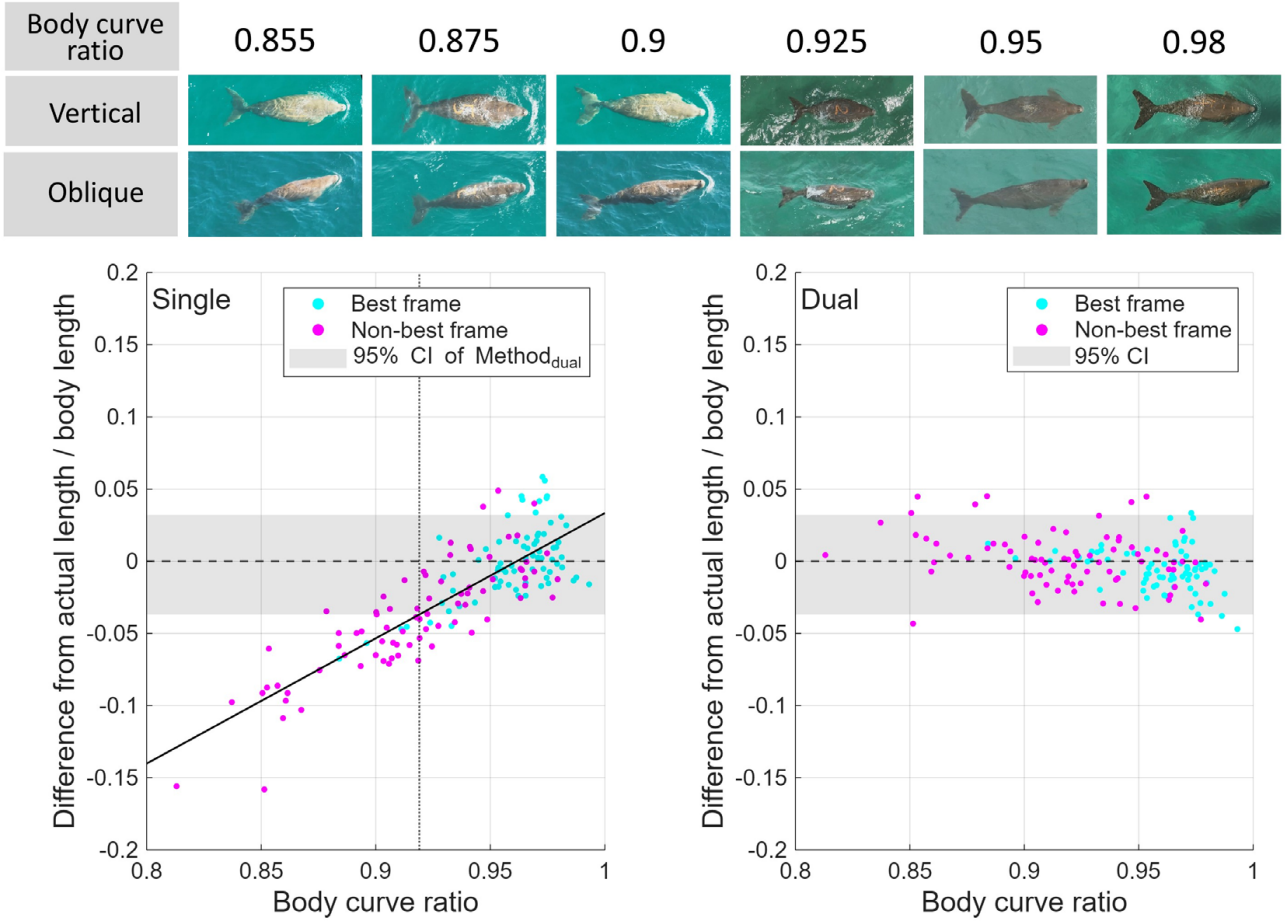
Estimation error relative to actual body length and body curve ratio. Examples from animals with known body curve ratios are shown at the top. The dashed vertical line in the left inset indicates the body curve ratio threshold of 0.919 at which the magnitude of estimation error of BL_single_ exceeded the lower limit of 95% CI of BL_dual_.

The mean body curve ratio of free‐swimming animals was 0.926 ± 0.049 (0.751–0.992; *n* = 188). Among the 188 animals examined, 31.9% had a body curve ratio of less than 0.919, the threshold at which the magnitude of estimation error of BL_single_ exceeded the lower limit of 95% CIs of BL_dual_ (Figure 10). Individuals with various body lengths were found to exhibit lower ratios in some cases.

**FIGURE 10:**
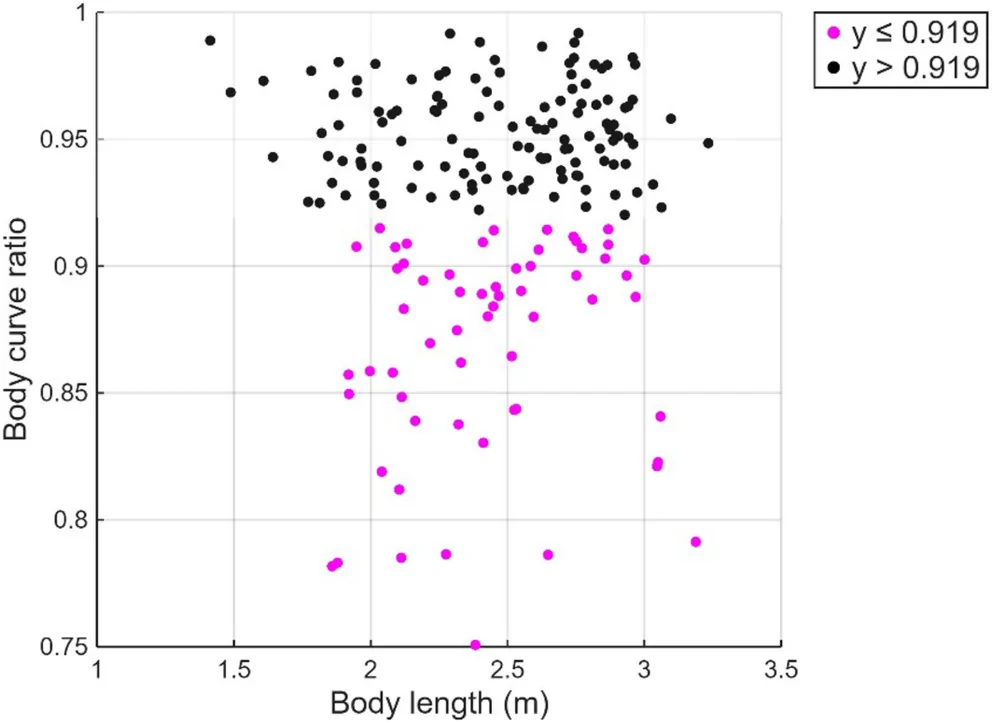
Body curve ratio (y‐axis) and body length (x‐axis) of dugongs estimated using drone photogrammetry (BL_dual_). The pink point indicates an animal exhibiting strong body curvature, for which Method_dual_ is expected to outperform Method_single_.

## 4 Discussion

### 4.1 Accuracy of Body Length Measures

To our knowledge, this study is the first attempt to estimate the three‐dimensional posture of aquatic wildlife from the air, and is among the few that have evaluated the accuracy of total length estimation in free‐swimming aquatic mammals (Ramos et al. 2022; Vivier et al. 2023). The estimation accuracy of the proposed method (3.6 ± 3.0 cm, or 1.4% ± 1.2% of body length; GSD = 2.4 mm) for dugongs with various body poses is better than measurements of a manatee using a LiDAR‐mounted drone and posture‐filtered images (−0.19, −0.16, and −0.03 m, or 6.1%, 5.1%, and 0.1%; estimated GSD = 10–23 mm/pixel; Ramos et al. 2022) and comparable to total body length estimates of dolphins based on blowhole‐to‐dorsal fin‐length (BHDF) in previous studies (8 ± 8 cm or 3.3% ± 3.1%; estimated GSD = 2–7 mm/pixel, assuming a focal length of 25 mm; Vivier et al. 2023). The finer GSD in this study may have partly contributed to the higher estimation accuracy than that reported by Ramos et al. (2022). Vivier et al. (2023) assigned dolphins to three age‐class categories (calves, juveniles, and adults) with 62%–83.8% accuracy. Similarly, distinguishing among dugong adults (> 260 cm), subadults (200–240 cm), and calves (< 200 cm) requires resolving size bins of 40–50 cm (Burgess, Lanyon, Brown, et al. 2012; Burgess, Lanyon, and Keeley 2012). The accuracy of our method is therefore likely sufficient to reliably assign dugongs to these size/maturity classes.

The estimation accuracy of the proposed method was comparable to those that have been achieved for stationary animals or objects in some cases. Using the same equipment and animals as above, Vivier et al. (2023) estimated the BHDF‐derived total body length of stationary dolphins which were supported by husbandry staff under the caudal region to maintain the body straight and the fluke flat. The estimation error was 0 ± 3 cm or 0.1% ± 1.3%, highlighting how body curvature during movement can introduce measurement errors. Krause et al. (2017) estimated total body length of nine hauled‐out leopard seals (3 ± 0.02 m) with an estimation accuracy of 6 ± 3 cm or 2.01% ± 1.06%, using a non‐LiDAR drone and a reference object placed near the target animal for scale calibration (estimated GSD = 2–4 mm/pixel; flight altitude = 23–45 m; Krause et al. 2017). Comparable accuracy has been reported for floating objects: 6.9 ± 1.2 cm or 3.57% ± 0.62% for a dolphin replica (1.95 m) using a non‐LiDAR drone (estimated GSD = 6–23 mm/pixel; flight altitude = 15–60 m; Christie et al. 2022), and 2.7 ± 4.9 cm for a floating object (2.7 m) using a LiDAR drone (estimated GSD = 2–5 mm/pixel; flight altitude = 16–30 m; Dawson et al. 2017).

### 4.2 Precision of Body Length Measures

A number of studies have reported the precision of total body length measurements obtained from repeated measurements of animals with unknown true length, often expressed as the coefficient of variation (CV; SD/mean): 1.2% (≈15 cm) for southern right whales ( *Eubalaena australis* ; total body length: 10.4–14.0 m) (Dawson et al. 2017); 4.8% ± 3.7% for lactating females and 3.1% ± 2.2% for calves of southern right whales (Christiansen et al. 2018); 2.2% for blue whales ( *Balaenoptera musculus* , Burnett et al. 2019); 3% (≈6 cm) for Australian snubfin dolphins ( *Orcaella heinsohni* , 2.07 ± 0.23 m) and humpback dolphins ( *Sousa sahulensis* , 2.04 ± 0.35 m) (Christie et al. 2022); 3.8% for bottlenose dolphins (1.4–3.4 m; Cheney et al. 2022). In bottlenose dolphins, the CVs of body widths measured at 20%–60% of body length (≤ 50 cm) ranged from 0.12% to 14.9% (Cheney et al. 2022). Most of these studies excluded images in which animals exhibited strong body curvature, and some studies further excluded data with CV values exceeding thresholds (Burnett et al. 2019; Christiansen et al. 2016). Given that models based on filtered data successfully detected among‐ and within‐population (e.g., intra‐seasonal) differences in body condition in baleen whales (Christiansen et al. 2016, 2018, 2020), a CV of < 5% as reported in these studies may be acceptable for addressing certain ecological questions (e.g., energetic costs associated with growth and reproduction; nutritional status of individuals and populations and its determinants). Mean interquartile range of BL estimation errors within individual across three dives, and best and non‐best frames was 4.4 ± 1.7 cm, or 1.7% ± 0.7% of body length, and comparable to these studies.

Overall, both accuracy and precision of our method using dual drones were comparable to those reported in these previous and highly controlled studies. It is noteworthy that our accuracy in measuring body lengths of free‐swimming dugongs is comparable to that obtained for stationary animals/objects, despite the animals showing various body postures and when surface waters were disturbed.

### 4.3 Error Sources

Known sources of error in drone photogrammetry include inaccurate altitude readings from drone or laser, obscured body extremities, annotation errors, water distortion, angle of the camera and lens distortion, and body curvature (Dawson et al. 2017; Bierlich, Schick, et al. 2021; Christie et al. 2022; Vivier et al. 2023; Segre 2025). Among body curvature, obscured body extremities, flight altitude, position within the image frame, and body roll, body curvature was considered the factor most strongly associated with the precision of body length measurements in snubfin dolphins (Christie et al. 2022).

Method_dual_ is likely less susceptible than Method_single_ to the posture‐dependent errors (i.e., overestimation of snout–nostril length when the animal faces upward, underestimation of peduncle–fluke notch length during fluke strokes, and underestimation of overall body length with increasing body curvature). Unlike Method_single_, Method_dual_ showed no systematic directional change in estimation error either (1) when the body extremities used to define body length were changed (Figure 5) or (2) when the degree of body curvature increased (Figure 9).

In our study, fluctuations in drone altitude readings were unlikely to have caused substantial scaling error. The SD of the Camera V altitude readings corresponded to 0.068% ± 0.083% and 0.13% ± 0.2% of the 40‐m flight height over *t* ± 2 s and 10‐s intervals, respectively, and differences between maximum and minimum altitude readings corresponded to 0.24% ± 0.3% and 0.47% ± 0.69% of the flight height. Furthermore, these fluctuations do not necessarily represent uncertainty in the altitude readings, because slight actual ascents or descents may have occurred when the operator inadvertently adjusted the control sticks during the 4‐s and 10‐s intervals. Inaccuracy in drone altitude was addressed by combining the use of a reference object for measurement validation, and with drones capable of precise measurement of altitude.

This study suggests that clarity of body extremities is associated with measurement accuracy, as suggested by Christie et al. (2022). In this study, a few centimeters of among‐frame variance in apparent fluke width were likely due to water movement. It was not feasible to average measurements from consecutive frames since the visibility of extremities varied among frames due to water ripples, splashes, and camera focus. Thus, a few centimeters of imprecision may be unavoidable in body length measurements of aquatic animals in natural environments.

Minor errors may have occurred in on‐deck measurements, although out‐of‐water measurements of stationary animals are generally the most accurate. Minor errors in straight body length due to body curvature along the horizontal plane were likely inevitable for some animals, although several personnel repositioned the animal to ensure a straight posture before taking measurements. Peduncle–fluke notch length was affected by uncertainty in identifying peduncle location, as described in Section 2.1. In drone footage, peduncle location was also difficult to define because ripples often obscured the body contour. Consequently, the use of the peduncle as an extremity is not recommended. Minor errors might have occurred in fluke width measurements in those infrequent cases when a fluke tip was absent and thus the exact measurement points differed among observers. Measurements of curved snout–nostril and peduncle–fluke notch lengths were also susceptible to errors in the 3D models. Another potential source of error is uncertainty in body dorsal line annotation. Although the body dorsal line was clearly marked out‐of‐water, all of the marks were not always clearly visible in water.

Our linear mixed‐effects model of estimation error indicated that Method_dual_ tended to underestimate body length of the animal with straight body posture, but the underlying cause is yet to be determined, as the variation in error was also substantial (Figure 6, upper‐left inset; Table 1). One possible explanation is that a more horizontal body posture tends to cause greater surface disturbance, which may obscure body extremities. Estimation errors across different body length definitions (Figure 5) suggested that errors in Method_single_ also include overestimation of snout‐nostril length when the animal faces upwards and tilts its snout up and forwards, and underestimation of peduncle‐fluke notch length, possibly during tail thrusts.

One possible error source specific to Method_dual_ is inaccuracy in the camera orientation measurements, which affects the z‐dimension (depth) of reconstructed 3D coordinates of body dorsal line. Whereas laboratory‐based 3D reconstructions typically determine the camera viewing direction by empirically filming a checkerboard (Karashchuk et al. 2021), our approach relies on the camera orientations recorded in flight logs. Only a few studies have validated orientation error of consumer‐grade drones under field conditions, likely due to the difficulty in obtaining true values. A field study using a DJI M2P reported yaw errors mostly of 1°–2°, with an occasional error of 20° (Watts et al. 2024). In addition, latitude and longitude were logged at sub‐meter‐level resolution, which may have contributed to minor error in calculating the camera O‐to‐object distance.

### 4.4 Potential Contribution of the Dual Drone Method

The proposed method using dual drones (positioned vertically and obliquely relative to the animal) enables data collection from animals and dives that would otherwise have been discarded as poor quality. Being able to sample from almost every animal and every surfacing event is particularly useful when the encounter rate is low, the animal is visible near the surface momentarily (i.e., when it is unrealistic to observe multiple breaths), and duration of fieldwork is limited. This method not only improves success rate in measuring body size of a free‐swimming animal but also helps ensure unbiased sampling and allows for rapid data collection. It has been demonstrated that under usual conditions, a substantial proportion of free‐swimming dugongs maintained markedly curved postures. The proportion of images remaining after the visual image quality control was 57% for dugongs in Thailand (Yamato et al. 2024), 94% for gray whales (Lemos et al. 2020), 38% for southern right whales (Christiansen et al. 2018), and 67% for humpback whales ( *Megaptera novaeangliae* , Christiansen et al. 2016).

The proposed method provides an objective metric of posture such as body curve ratio. Existing studies have generally determined data inclusion based on visual inspection of vertical shots only. Nevertheless, accurately assessing body curve ratio, and thus the associated estimation error in Method_single_, based on visual inspection of vertical shots only, appears challenging. The use of an objective indicator is valuable for defining thresholds for data exclusion (thereby clarifying data reliability).

This system was designed for measuring dugongs, but has the potential to be applied to a range of air‐breathing aquatic megafauna. In particular, it will enhance data collection for species lacking distinct features along the body dorsal line (e.g., other sea cows, finless porpoises, belugas, and right whales which do not have dorsal fins) and enable 3D analysis of surfacing behavior across a broader range of taxa.

### 4.5 Limitations of the Dual Drone Method

As with any body measurements, there must be obvious landmarks, and these must be fixed and visible. In aquatic fauna, the visibility is affected by environmental conditions (e.g., turbidity, glare, sea state, and water depth) and by an animal's behavior (e.g., surfacing posture, disturbance of water). When measuring animals with varying postures, the visibility of body extremities becomes the primary constraint on expanding data collection. In our study site, there was always a moment during each surfacing event when body extremities were visible, but this may not be the case in more turbid waters. The oblique footage also must capture both body extremities. In the case of dugongs, when an animal turned whilst surfacing, the snout was not always visible. This occasionally limited the availability of usable data.

The cost of introducing two drones is a second limitation. A LiDAR unit and the drone set commonly used in marine mammal studies costs approximately US$5640 in 2017 (Dawson et al. 2017). The official price of our drone set and a GNSS receiver was approximately US$4200 and US$390, respectively. Thus, using two drone sets would cost about US$8800 in total (2025 prices). To further reduce costs and applicability, it is yet to be assessed whether the RTK drone could be replaced by a LiDAR drone or a non‐LiDAR drone.

Obtaining oblique footage requires a certain level of operational proficiency, since the operator must simultaneously control the drone's horizontal movement and yaw direction to keep it to the side of the animal, whereas obtaining vertical footage requires only control of a drone's horizontal movement to keep it above the animal. In this study, oblique filming was performed by one of the two operators with more than several hundred hours of flight experience, whereas vertical footage was obtained by one of the five operators with little more than a few hours of flight experience. Valid data were obtained from all animals with all combinations of pilots. Considering that the proposed method substantially improved the success rate and efficiency of data collection, the benefits may outweigh these costs.

Adding a drone may impose an additional risk of affecting animals. Compared with high‐altitude flights, low‐altitude drone flights (below 30 m) are likely to provoke more disturbance reactions among cetaceans (Aubin et al. 2023). We currently use protocols regarded to have minimal impact on the animals (40 m), including dugongs, but these should be refined as future evidence on animal responses becomes available. In our study, 15 of randomly sampled 188 animals surfaced close to the water surface without breathing 1.4 ± 0.7 times out of 9.1 ± 3.2 surfacing events, which may share similarity with the decreased respiration rates observed in drone‐observed manatees (Landeo‐Yauri et al. 2021). One of the 188 animals showed a sudden dive. We did not observe other apparent evasive or alert behaviors commonly described for cetaceans, nor fleeing, grouping, or tail‐kicks reported for manatees (Landeo‐Yauri et al. 2021; Ramos et al. 2018).

## 5 Conclusion

This study demonstrated that varying posture can cause substantial errors in aerial body size measurements in free‐ranging marine megafauna; however, such errors can be minimized through methodological adjustments. It not only improves the success rate in obtaining accurate body measurements but also helps ensure unbiased sampling and allows for rapid data collection.

## Author Contributions

**Chiaki Yamato:** conceptualization (lead), data curation (equal), formal analysis (lead), funding acquisition (lead), investigation (equal), methodology (equal), project administration (equal), resources (equal), software (lead), validation (equal), visualization (lead), writing – original draft (equal), writing – review and editing (equal). **Helen L. Sneath:** data curation (equal), investigation (equal), project administration (equal), resources (equal), supervision (equal), writing – review and editing (equal). **Janet M. Lanyon:** conceptualization (equal), data curation (equal), formal analysis (equal), investigation (equal), project administration (lead), resources (equal), validation (equal), writing – review and editing (equal).

## Funding

This work was funded by JSPS (Japan Society for Promotion of Science) overseas research fellowship and Sea World Foundation. UQ Moreton Bay Research Station provided boating support.

## Conflicts of Interest

The authors declare no conflicts of interest.

## Figures and Tables

**FIGURE A1:**
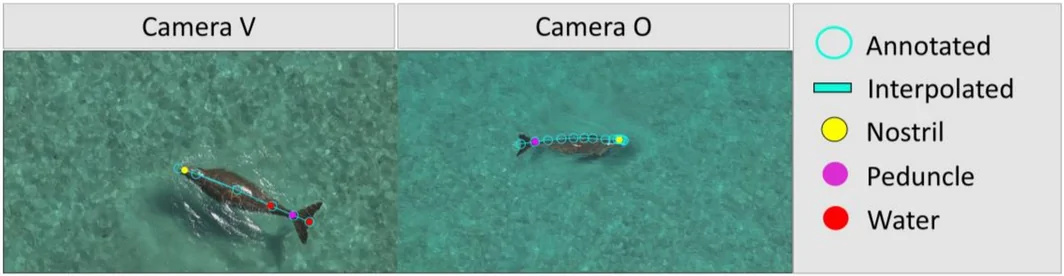
Example of annotated and interpolated points. The cyan line in the left inset represents the interpolated points $V_{\text{interpolated}\_x_{i}},V_{\text{interpolated}\_y_{i}}$, while the cyan line in the right inset represents $O_{\text{interpolated}\_x_{i}},O_{\text{interpolated}\_y_{i}}$.

**FIGURE A2:**
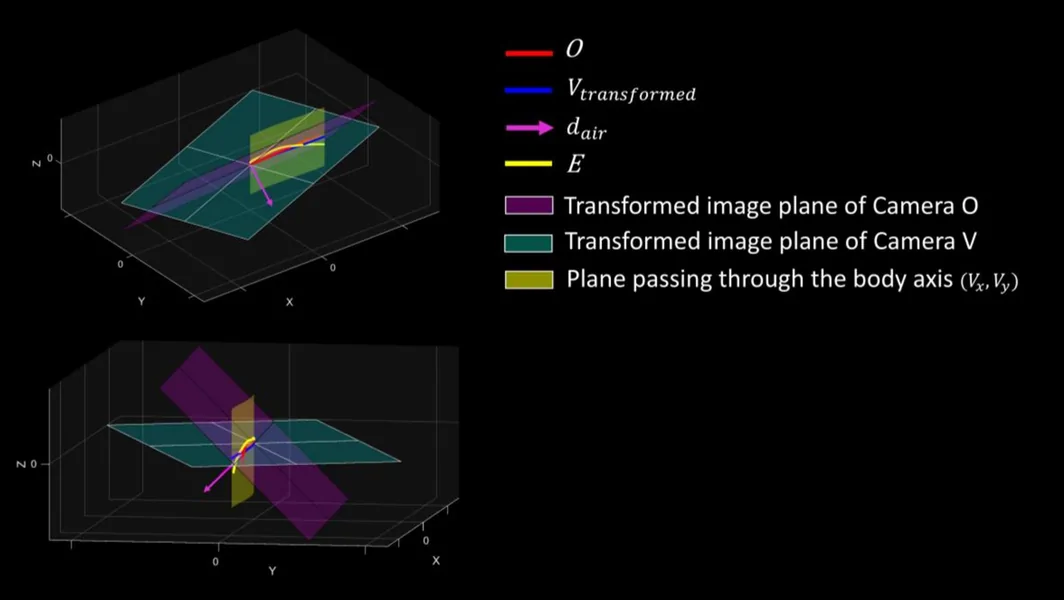
Illustration of the transformed image planes, transformed image coordinates of the dorsal line, Camera O's viewing direction in air, and the resulting estimated 3D coordinates of the dorsal line. The same data shown in Figure A1 is used for this example. Camera V faces the negative z‐axis.

**FIGURE A3:**
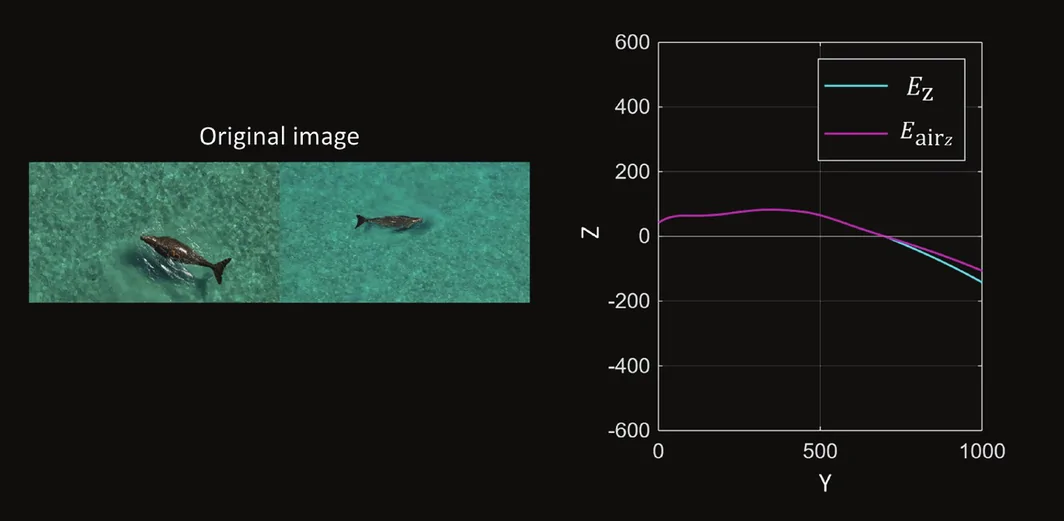
Example of apparent 3D coordinates of the body dorsal line $E_{\mathrm{air}}$ without assuming a water refraction and corrected 3D coordinates E. The same data shown in Figure A1 is used for this example.

## Data Availability

Scripts and data available from Figshare Repository (https://figshare.com/s/4eba3cecc208d95a280f).

## Appendix A

### Estimation of 3D Coordinates of Body Dorsal Line

The 3D coordinates of the body dorsal line were estimated by tracing rays from the drone camera positions using camera orientations and positions recorded in the flight log. The intersection of the viewing direction of Camera O with the plane passing through the body dorsal line seen from Camera V was first computed under the assumption of straight light paths in air. For underwater portions of the body dorsal line, the 3D coordinates of the body dorsal line were corrected for water‐surface refraction by projecting each apparent point onto the water plane and computing the refracted ray direction using Snell's law. The pixel length was converted into an actual length using (1) the real‐world and pixel lengths of the reference object filmed before and after each flight, (2) the real‐world and pixel lengths of a reference object filmed from a known distance, and (3) the drone altitude recorded at the time of the reference measurement and body measurement.

#### Before Field Data Collection

##### A1. Ground Sampling Distance

Ground Sampling Distance (GSD) is the ratio of distance in real world (e.g., meters) to distance in the image (pixels). It is commonly calculated using the camera sensor size and the focal length of the lens (Torres and Bierlich 2020), or by modeling relationship between altitude readings and pixel length of an object with known real‐world length (Burnett et al. 2019). Segre (2025) pointed out that using manufacturer‐provided camera parameters to calculate GSD can be problematic for some consumer drones due to the internal image processing used to mitigate lens distortion (Segre 2025). Because the internal image processing used in our drone model and zoom settings was unknown, we empirically determined the GSD with the model and setting used in this study (Mavic 3E, 7× zoom). An object with known length ${\mathrm{Ref}}_{\mathrm{pre}\_\mathrm{act}}$ (i.e., two points on a checkerboard which were 0.339 m apart) and the drone were placed on the ground. The distance between the drone and the object ${\mathrm{Alt}}_{\mathrm{pre}}$ (5 m in this study) was measured with a steel tape measure. We selected 5 m as the distance that could be accurately measured to the nearest millimeter with a steel tape measure, and 0.339 m as the object length because it occupied approximately one‐third of the image width at 7× zoom. Video was taken with 7× zoom, and the pixel length ${\mathrm{Ref}}_{\mathrm{pre}\_\mathrm{pix}}$ of the object were measured. We assumed a 1:1 proportional relationship between increments in altitude calculated using Real‐Time Kinematic measurements and pixel distance, while the built‐in barometers of consumer‐grade drones are known to show deviations from a perfect 1:1 relationship (Segre 2025).

##### A2. Lens Distortion Correction

Image distortion (barrel distortion) was corrected using the Single Camera Calibrator App in MATLAB R 2025a. We captured a video while rotating a checkerboard at various angles with 7× zoom. Frames were extracted from the video and ingested into the application to estimate the camera‐specific intrinsic parameters. Each frame used in subsequent analysis was corrected for lens distortion using these parameters prior to annotation.

#### After Field Data Collection

##### A3. Calibration

Camera V's video of the reference object (i.e., a vessel duckboard or a buoy) was reviewed frame by frame to identify the frame with optimal visibility of extremities. The drone's altitude as this timing (r) was extracted from the flight log. The two extremities of the reference object with known actual length ${\mathrm{Ref}}_{\mathrm{act}}$ were manually annotated in the image, and the corresponding pixel distance between the extremities (${\mathrm{Ref}}_{\mathrm{pix}}$) was measured. The altitudinal distance between the Camera V and the reference object was then estimated using the parameters ${\mathrm{Alt}}_{\mathrm{pre}}$, ${\mathrm{Ref}}_{\mathrm{pre}\_\mathrm{pix}}$, and ${\mathrm{Ref}}_{\mathrm{pre}\_\mathrm{act}}$ obtained before the field data collection, according to:

$${\mathrm{Alt}}_{\text{camV}\_{\mathrm{est}}_{\left(r\right)}}={\mathrm{Alt}}_{\mathrm{pre}}\times \frac{\;{\mathrm{Ref}}_{\mathrm{pre}\_\mathrm{pix}}}{\;{\mathrm{Ref}}_{\mathrm{pre}\_\mathrm{act}}}\times \frac{\;{\mathrm{Ref}}_{\mathrm{act}}}{\;{\mathrm{Ref}}_{\mathrm{pix}}}$$

${\mathrm{Alt}}_{\text{camV}\_{\mathrm{est}}_{\left(r\right)}}$ was calculated for each flight to compensate for the unknown launch height. ${\mathrm{Ref}}_{\mathrm{pre}\_\mathrm{act}}$ divided by ${\mathrm{Ref}}_{\mathrm{pre}\_\mathrm{pix}}$ represents the empirical GSD, which was 2.4 mm/pixel at 7× zoom for the drones used in this study. This value did not differ substantially from the GSD of 2 mm/pixel obtained through camera sensor specification provided by manufacturer (DJI 2025).

##### A4. Annotation and Interpolation

The synchronized video of the dugong's breath was reviewed frame by frame to identify the frame with optimal visibility of the extremities. As this timing (t), the following features were annotated (see Figure A1 for example):

1. The central body dorsal lines in the vertical and oblique frames.
2. The boundary between the in‐water and out‐of‐water area along the body dorsal line
3. Nostril and peduncles in the vertical and oblique frames, if body lengths for different definitions were to be measured
4. Fluke tips in the vertical frame, if fluke width was to be measured

Spline interpolation was performed to the annotated image coordinates to generate evenly spaced points $V_{\text{interplated}\_x_{i}},V_{\text{interpolated}\_y_{i}}$ for vertical shot and $O_{\text{interpolated}\_x_{i}},O_{\text{interpolated}\_y_{i}}$ for oblique shot $\left(i=1,\ldots ,1000\right)$ (Figure A1).

##### A5. 3D Coordinate Estimation

1. The drone cameras' attitude parameters (gimbal yaw, gimbal pitch, latitude, longitude, and altitude) at the timing t were extracted from the flight log. Gimbal roll was not considered in this study because it was always 0 in most flight logs checked preliminary. Latitude and longitude are provided in the UTM (Universal Transverse Mercator) coordinate system (unit: meter).
2. The image planes and interpolated coordinates of Cameras V and O ($V_{\text{interpolated}\_x_{i}},V_{\text{interpolated}\_y_{i}}\;\text{and}\;O_{\text{interpolated}\_x_{i}},O_{\text{interpolated}\_y_{i}}$) were transformed such that (1) the starting positions of the body dorsal lines $\left(V_{x_{1}},V_{y_{1}}\right)\;\text{and}\;\left(O_{\text{transformed}\_x_{1}},O_{\text{transformed}\_y_{1}}\right)$ were aligned with the origin $\left(0,0,0\right)$, and (2) the direction vector between the start and end positions of the body dorsal line seen from the Camera V $\left(V_{x_{1000}},V_{y_{1000}}\right)$ is aligned along the y‐axis (see Figure A2 for example). The image plane is oriented along the camera's line of sight and the image coordinates are always lied on the image plane. The relative orientation of the cameras is preserved through the transformation.
3. The 3D coordinates of the dorsal line $O_{\text{transformed}}$ were scaled based on the ratio of the camera‐to‐target (dugong) distances (Camera O‐dugong distance relative to Camera V‐dugong distance). The distance between Camera V and the dugong as timing (t) was:

$$D_{{\text{camV}}_{\left(t\right)}}={\mathrm{Alt}}_{\mathrm{cam}{V_{\mathrm{est}}}_{\left(r\right)}}+\left({\mathrm{Alt}}_{{\text{camV}}_{\left(t\right)}}-{\mathrm{Alt}}_{{\text{camV}}_{\left(r\right)}}\right)$$

This equation assumes that the relative altitudes estimated during reference and breath filming are accurate.

The distance between Camera O and the dugong was:

$$D_{{\text{camO}}_{\left(t\right)}}=\sqrt{p_{x}^{2}+p_{y}^{2}+p_{z}^{2}},$$

where $p_{x},p_{y},p_{z}$ were the difference of longitude, latitude, and altitude between Cameras O and V as timing (t):

$$p_{x}=|{\text{longitude}}_{{\text{camO}}_{\left(t\right)}}-{\text{longitude}}_{{\text{camV}}_{\left(t\right)}}|$$

$$p_{y}=|{\text{latitude}}_{{\text{camO}}_{\left(t\right)}}-{\text{latitude}}_{{\text{camV}}_{\left(t\right)}}|$$

$$p_{z}=D_{{\text{camV}}_{\left(t\right)}}+\left({\mathrm{Alt}}_{{\text{camO}}_{\left(t\right)}}-{\mathrm{Alt}}_{{\text{camV}}_{\left(t\right)}}\right)$$

and the relative distance between the dugong and cameras was:

$$\text{Relative}\_\text{distance}=\frac{D_{{\text{camO}}_{\left(t\right)}}}{D_{{\text{camV}}_{\left(t\right)}}}$$

The scaled dorsal line was:

$$\left(O_{x},O_{y},O_{z}\right)=\text{Relative}\_\text{distance}\times O_{\text{transformed}}$$

4 Apparent 3D coordinates of the dorsal line $E_{\mathrm{air}}=\left(E_{\mathrm{air}x},E_{\mathrm{air}y},E_{\mathrm{air}z}\right)$ was calculated without assuming a water refraction. The intersections between the vector passing through the point $\left(O_{x},O_{y},O_{z}\right)$ in Camera O's viewing direction $d_{\mathrm{air}}=\left(\sin\left({\mathrm{yaw}}_{{\mathrm{rel}}_{\left(t\right)}}\right),\cos\left({\mathrm{yaw}}_{{\mathrm{rel}}_{\left(t\right)}}\right),\tan\left({\text{pitch}}_{{\mathrm{rel}}_{\left(t\right)}}\right)\right)$ and the plane passing through the body axis $\left(V_{x},V_{y}\right)$ as seen in Camera V (see Figure A2) was found. Here, ${\mathrm{yaw}}_{{\mathrm{rel}}_{\left(t\right)}}$ and ${\text{pitch}}_{{\mathrm{rel}}_{\left(t\right)}}$ were the yaw and pitch of Camera O as timing t after the transformation.
5 For underwater portion of $E_{\mathrm{air}}$ annotated in Section A4, the apparent 3D points were corrected for distortion by water refraction (see Figure A3 for example). $E_{\mathrm{air}}$ was projected along the Camera O's air‐side viewing direction $d_{\mathrm{air}}$. Assuming the water surface lies on the plane z=w with the upward normal vector $n=\left(0,0,1\right)$, the intersection S of this ray with the water surface was calculated by:

$$s_{\text{plane}}=-\frac{w-E_{\mathrm{air}z}}{d_{\mathrm{air}z}},S=E_{\mathrm{air}}+s_{\text{plane}}\;d_{\mathrm{air}}$$

where $s_{\text{plane}}$ was the scalar distance along the ray to the plane. w was the z‐coordinate of $E_{\mathrm{air}}$ at the boundary between the above‐water and underwater areas defined in Section 2. The apparent path length (the distance from the water surface to the apparent point) $E_{\mathrm{air}}$ along Camera O's air‐side viewing direction $d_{\mathrm{air}}$ was

$$t_{\mathrm{app}}=|s_{\text{plane}}|,$$

The refracted direction in water computed using Snell's law was:

$$d_{w}=\text{refract}\left(d_{\mathrm{air}},n,\eta \right),\eta =\frac{n_{1}}{n_{2}},$$

η was the ratio of refractive indices with $n_{1}=1.00029$ (air) and $n_{2}=1.339$ (sea water). The true optical path length was then scaled to

$$t_{\text{true}}=\frac{n_{2}}{n_{1}}\frac{\cos i}{\cos t}\;t_{\mathrm{app}},$$

and the corrected underwater point was

$$E=S+t_{\text{true}}\;d_{w}.$$

##### A6. Body Length Estimation

Pixel lengths of the body lengths based on Method_single_ and Method_dual_ were:

$${\mathrm{BL}}_{\text{single}\_\mathrm{pix}}=\sqrt{{V_{x_{i}}}^{2}+{V_{y_{i}}}^{2}+{V_{z_{i}}}^{2}}\;\left(i=1,\ldots ,1000\right)$$

$${\mathrm{BL}}_{\text{dual}\_\mathrm{pix}}=\sqrt{{E_{x_{i}}}^{2}+{E_{y_{i}}}^{2}+{E_{z_{i}}}^{2}}\;\left(i=1,\ldots ,1000\right)$$

Actual body lengths and body curve ratio were:

$${\mathrm{BL}}_{\text{single}}={\mathrm{Ref}}_{\mathrm{act}}\times \frac{{\mathrm{BL}}_{\text{single}\_\mathrm{pix}}}{{\mathrm{Ref}}_{\mathrm{pix}}}\times \frac{{\mathrm{Alt}}_{{\text{camV}}_{\left(t\right)}}}{{\mathrm{Alt}}_{{\text{camV}}_{\left(r\right)}}}$$

$${\mathrm{BL}}_{\text{dual}}={\mathrm{BL}}_{\text{single}}\times \frac{{\mathrm{BL}}_{\text{singl}e_{\mathrm{pix}}}}{{\mathrm{BL}}_{{\text{dual}}_{\mathrm{pix}}}}$$

$$\text{Body curve ratio}=\frac{{\mathrm{BL}}_{\text{single}\_\mathrm{pix}}}{{\mathrm{BL}}_{\text{dual}\_\mathrm{pix}}}$$

**TABLE A1:** Dugong body length measurement errors for Method_single_ and Method_dual_, and paired *t*‐test results. Absolute error (%) denotes the error relative to the body lengths of each dugong measured on deck.

Method	Dataset	RMSE (m)	Absolute error (m) Mean ± SD [Range]	Absolute error (%) Mean ± SD [Range]	Difference between methods	
					*t*	*p*
Dual	Best (*n* = 75)	0.043	0.033 ± 0.028 [0.000–0.123]	1.3 ± 1.1 [0.0–4.9]	−2.95	0.0042
	Non‐best (*n* = 78)	0.05	0.039 ± 0.033 [0.000–0.117]	1.5 ± 1.2 [0.0–4.5]	−7.8	2.4 × 10^−11^
	All (*n* = 153)	0.047	0.036 ± 0.030 [0.000–0.123]	1.4 ± 1.2 [0.0–4.9]	−5.67	1.2 × 10^−7^
Single	Best (*n* = 75)	0.065	0.050 ± 0.042 [0.002–0.193]	2.0 ± 1.6 [0.1–7.2]		
	Non‐best (*n* = 78)	0.142	0.114 ± 0.084 [0.001–0.376]	4.8 ± 3.8 [0.1–18.8]		
	All (*n* = 153)	0.111	0.083 ± 0.054 [0.001–0.376]	3.4 ± 3.2 [0.1–18.8]		

**TABLE A2:** Summary of absolute estimation error (m) for dugong body length measures using Method_single_ and Method_dual_.

Dataset	Method	Mean ± SD/RMSE			
		Snout‐Fluke notch	Nostril‐Fluke notch	Nostil‐Peduncle	Snout‐Peduncle
Best (*n* = 75)	Single	0.050 ± 0.042/0.065	0.088 ± 0.059/0.106	0.070 ± 0.053/0.087	0.048 ± 0.038/0.061
	Dual	0.033 ± 0.028/0.043	0.039 ± 0.029/0.049	0.043 ± 0.034/0.055	0.039 ± 0.028/0.048
Non‐best (*n* = 29)	Single	0.097 ± 0.063/0.115	0.179 ± 0.066/0.190	0.134 ± 0.064/0.148	0.067 ± 0.050/0.083
	Dual	0.037 ± 0.034/0.050	0.047 ± 0.029/0.055	0.047 ± 0.029/0.055	0.042 ± 0.028/0.050
